# Two detoxification enzyme genes, *CYP6DA2* and *CarFE4*, mediate the susceptibility to afidopyropen in *Semiaphis heraclei*

**DOI:** 10.3389/fphys.2024.1478869

**Published:** 2024-12-06

**Authors:** Xiaochen Fu, Chao Xue, Xin Wang, Aiyu Wang, Yanwei Zhu, Yuanxue Yang, Yun Zhang, Yun Zhou, Ming Zhao, Chenggang Shan, Jianhua Zhang

**Affiliations:** ^1^ School of Pharmacy, Shandong University of Traditional Chinese Medicine, Jinan, China; ^2^ Institute of Industrial Crops, Shandong Academy of Agricultural Sciences, Jinan, China; ^3^ Chongqing Jiulongpo District Agro-Tech Extension and Service Station, Chongqing, China

**Keywords:** *Semiaphis heraclei*, afidopyropen, detoxification mechanism, *CYP6DA2*, *CarFE4*

## Abstract

**Introduction:**

*Semiaphis heraclei* is an important economic pest affecting Caprifoliaceae and Apiaceae plants, and chemical control is still the main effective control method in the field. Afidopyropen is a new type of pyridine cyclopropyl insecticide, which can effectively control piercing-sucking mouthparts pests and is suitable for pest resistance management. However, the detoxification mechanism of *S*. *heraclei* to afidopyropen is still poorly cleared.

**Methods:**

The insecticidal activity of afidopyropen against *S. heraclei* and the enzyme activity assay and synergism bioassay were evaluated. The detoxification enzyme genes were obtained by transcriptome and validated by quantitative real-time PCR (RT-qPCR). Furthermore, RNA interference was used to study the functions of detoxification enzyme genes.

**Results:**

The activities of cytochrome P450 monooxygenases (P450s) and carboxylesterases (CarEs) were significantly increased under afidopyropen treatment. The toxicity of afidopyropen against *S. heraclei* was significantly increased after application the inhibitors of piperonyl butoxide and triphenyl phosphate. Sixteen P450 genes and three CarE genes were identified in the transcriptome of *S. heraclei*. The RT-qPCR results showed that eleven P450 genes and two CarE genes were significantly upregulated under afidopyropen treatment, and the expression of *CYP6DA2* and *CarFE4* was upregulated by more than 2.5 times. The expression pattern of *CYP6DA2* and *CarFE4* was further analyzed in different developmental stages of *S. heraclei* and knockdown of *CYP6DA2* and *CarFE4* significantly increased the susceptibility of *S. heraclei* to afidopyropen.

**Conclusion:**

The results of this study uncover the key functions of *CYP6DA2* and *CarFE4* in the detoxification mechanism of *S. heraclei* to afidopyropen, and provide a theoretical basis for the scientific use of afidopyropen in the field.

## 1 Introduction

The *Semiaphis heraclei*, also known as celery aphid, is a Hemiptera insect widely distributed in the Far East and the Indian Subcontinent. The main hosts are Caprifoliaceae plants from Japan, China and East Siberia. The secondary hosts are Apiaceae plants from East and Southeast Asia, India and Pakistan, Algiers and Hawaii (Song et al., 2023). The *S. heraclei* will congregate on fresh stems, leaves, flower buds, and other parts of the host plant, where they feed on the plant by sucking the sap through their mouthparts. A significant infestation of *S. heraclei* can result in the curling of leaves, which impairs photosynthesis and ultimately leads to the yellowing or even wilting of the leaves. Additionally, they can affect the bud stage, which further impacts the growth of the plant and the content of active ingredients. In addition, the honeydew of *S. heraclei* can induce soot disease (Massimino Cocuzza, 2022). Moreover, the outbreak period of *S. heraclei* and harvest period of host plants such as honeysuckle are basically the same, which causes serious yield loss or even no yield to the honeysuckle, celery and other herbal vegetables (Song et al., 2023).

In order to reduce the impact of agricultural pests on field production, a series of field control methods are usually used, including physical and biological control. However, chemical insecticide control is still the most dependent method because of its quick effect and simple operation (Gregg et al., 2018; Mubeen et al., 2023). In recent years, as the use of pesticides increases, problems such as pesticide residues and environmental pollution become more serious. This poses a major risk to human health and to the safety of the food supply and has become a public health issue of great concern (He et al., 2023). In particular, fields require multiple applications of chemical insecticides to ensure crop and herb yields. This can easily lead to problems of excessive pesticide residues. In order to guarantee the safety of food and medication, the use of highly effective insecticides that are less harmful to natural enemies and the environment is key to the scientific control and management of pests in the field.

Afidopyropen is a derivative of pyripyropene A produced by filamentous fungus *Penicillium coprobium*. It is currently used worldwide as an alternative to traditional insecticides. It is particularly effective against aphids such as *Myzus persicae*, *Aphis gossypii* and *Aphis craccivora* (Goto et al., 2019). Afidopyropen quickly interferes with the feeding direction of insects by targeting transient receptor potential vanilloid (TRPV) cation channels. This weakens the ability of insects to coordinate movement, sense sound, maintain position and feed on plants, and ultimately lead to starvation and death (Jeschke, 2021; Yin et al., 2024). Although its insecticidal spectrum is narrow, it has high selectivity and insecticidal efficacy against target pests, low toxicity to natural enemies such as bees, seven-star lady beetles and mammals, and short persistence in crops and environment. In addition, afidopyropen can penetrate from treated to untreated leaves. This significantly improves insecticidal efficacy and reduces the amount of insecticide used (Horikoshi et al., 2022; Liu X. et al., 2024). These benefits are indicated that afidopyropen is a valuable sustainable agricultural green insecticide and an optimal agent for aphid control.

Exogenous detoxification and metabolism are the main physiological processes after insecticide exposure (Lu et al., 2021). The detoxification of insecticides in insects can be divided into three phases. Cytochrome P450 monooxygenases (P450s) and carboxylesterases (CarEs) are directly involved in the metabolism of exogenous substances as enzymes of phase I reaction (Li P. R. et al., 2023). Glutathione S-transferases (GSTs) are involved in the phase II reaction, via catalytic reaction to increase the water solubility of exogenous substances. In phase III reaction, water-soluble compounds are transferred and excreted out of the cell (Yang B. et al., 2020; Aioub and Ashour, 2023).

In this study, we evaluated the insecticidal activity of afidopyropen against the *S. heraclei*. Enzyme activity assay and synergism bioassay showed that P450s and CarEs participated in the detoxification of afidopyropen. The differentially expressed genes of P450s and CarEs were determined via transcriptome sequencing and their relative transcript levels were validated by quantitative real-time PCR (RT-qPCR). Furthermore, the functions of *CYP6DA2* and *CarFE4* in the susceptibility of *S. heraclei* to afidopyropen were determined by RNA interference (RNAi). The results of this study will lay a foundation for further research on *S. heraclei* and provide a theoretical basis for the field control of aphid and the rational use of afidopyropen.

## 2 Materials and methods

### 2.1 Insects and chemical reagents

The *S. heraclei* population was collected from Pingyi (35.60°N, 117.75°E; Linyi, China) in 2024. The *S. heraclei* was reared with celery in the laboratory at 25°C ± 1°C with 70%–80% relative humidity and 16 h light: 8 h dark photoperiod.

Afidopyropen (94.00%) was purchased from Shanghai Yuanye Biotechnology Co., Ltd (Shanghai, China). Acetone, dimethyl sulfoxide (DMSO), piperonyl butoxide (PBO), triphenyl phosphate (TPP) and diethyl maleate (DEM) were purchased from Merck KGaA (Darmstadt, Germany).

### 2.2 Bioassay

The bioassay was carried out by the topical application method via MICRO2T SMARTouchTM device (SMARTouchTM Controller for UMP3/Nanoliter 2010; World Precision Instruments, Inc.). Afidopyropen was dissolved in DMSO and then diluted with acetone to generate five concentrations. The third-instar nymphs of *S. heraclei* were anesthetized by ice and a droplet solution of 50 nL was applied topically to the dorsal plate. The nymphs treated with acetone were as the control. The treated insects were fed with celery at 25°C ± 1°C with 70%–80% relative humidity and 16 h light: 8 h dark photoperiod. Each treatment was repeated three times and contained thirty insects. Mortality was checked after 48 h.

### 2.3 Sample collection

The third-instar nymphs of *S. heraclei* treated with afidopyropen at the dose of median lethal dose (LD_50_), and the survival nymphs were collected at 48 h. The nymphs treated with acetone were as the control. The insects were immediately frozen in liquid nitrogen and then stored at −80°C for enzyme activity assay, transcriptome sequencing and quantitative validation. Each treatment was repeated three times and each contained thirty insects.

Besides, the first-to fourth-instar nymphs and the apterous adults of *S. heraclei* were collected for instar expression profile analysis. The insects were immediately frozen in liquid nitrogen and then preserved at −80°C. Each sample was repeated three times and contained thirty insects.

### 2.4 Enzyme activity assay

The protein concentration was determined by BCA protein quantitative/concentration determination kit provided by Beijing Labgic Technology Co. Ltd. (Beijing, China). The activities of P450, CarE and GST were determined by MFO activity assay kit, CarE activity assay kit and GST activity assay kit produced by Suzhou Comin Biotechnology Co., Ltd. (Suzhou, China).

Determination of P450 enzyme activity: according to the manufacturer’s instructions, approximately 0.1 g of the sample was taken and 1 mL of the extraction reagent was added and homogenized on ice, resulting in the production of a crude enzyme extract. The crude enzyme solution was centrifuged at 12,000 g for 20 min at 4°C. The supernatant was collected and the absorbance value of P450 was determined at 400 nm using a microplate reader with p-nitrophenol as the substrate. The production of 1 nmol of p-nitrophenol per milligram of histone per minute was defined as one unit of enzyme activity (Li Z. et al., 2023).

Determination of CarE enzyme activity: the crude enzyme solution was centrifuged at 12,000 g for 30 min at 4°C. The supernatant was collected and the absorbance value of CarE was determined at 450 nm using a microplate reader with α-naphthyl acetate as the substrate. Each milligram of histone is defined as 1 enzyme activity unit per minute increase in the catalytic absorbance value in the 37°C reaction system (Patra and Hath, 2023).

Determination of GST enzyme activity: the crude enzyme solution was centrifuged at 8,000 g for 10 min at 4°C. The supernatant was collected and the absorbance value of GST was determined at 340 nm using a microplate reader with 1-chloro2,4-dinitrobenzene (CDNB) as the substrate. At 37°C, each milligram of protein catalyses the binding of 1 nmol/L of CDNB to GSH as 1 unit of enzyme activity per minut (Cui et al., 2018; Hu et al., 2022).

BCA protein concentration assay: according to the manufacturer’s instructions, the BCA working solution was prepared. 20 μL of BCA protein standard solution (5 mg/mL) was diluted to 100 μL with PBS solution (0.01 mol L^−1^, pH 7.2) to give a final concentration of 1 mg/mL. The BCA standard assay solution was prepared in accordance with the instructions provided. The requisite volume of the sample to be measured was then added to the microtiter plate, and the solution was brought to a volume of 20 μL with PBS. A volume of 200 µL of the BCA working solution was added to the microtiter plate, which was then mixed thoroughly and left at 37°C for 30 min. The absorbance value at 562 nm was determined and documented, with the light absorption value of the BCA-free sample serving as the control. The protein concentration in the samples was calculated by constructing a standard curve with A562 as the vertical coordinate and BSA content as the horizontal coordinate.

### 2.5 Synergism bioassay

One hour before the application of afidopyropen, 2 μg of PBO, TPP and DEM in 50 nL acetone was applied topically to the dorsal plate of third-instar nymphs of *S. heraclei*. The nymphs treated with acetone were as the control. Then, the nymphs were anesthetized by ice and treated with afidopyropen at the dose of LD_50_ (Ding et al., 2013; Zhang et al., 2016; Yang B. et al., 2020). The treated insects were fed with celery at 25°C ± 1°C with 70%–80% relative humidity and 16 h light: 8 h dark photoperiod. Each treatment was repeated three times and contained thirty insects. Mortality was checked after 48 h.

### 2.6 RNA isolation, library construction and sequencing

Total RNA of the sample was extracted using TRIzol reagent (Invitrogen, United States) according to the manufacturer’s procedure. RNA purity, concentration and integrity were measured using a 5,300 Bioanalyzer (Agilent, United States) and an ND-2000 (NanoDrop Technolog, United States). Six *S. heraclei* RNA samples from the control group and the afidopyropen treatment group were taken for cDNA library construction and sequenced in BGI (Shenzhen, China).

### 2.7 Transcriptome assembly and function annotation

Before proceeding with the *Denovo* assembly, removed the reads containing joints (joint contamination) from the original reads and removed the reads with unknown base N content greater than 1%. Low-quality reads (those with a mass value of less than 15 bases accounting for more than 40% of the total base number of the reads were defined as low-quality reads) were removed to obtain clean reads. High-quality clean reads were *de novo* assembled using Trinity, followed by clustering of transcripts to de-redundancy using CD-HIT to obtain unigene. The assembled unigene was combined with Kyoto Encyclopedia of Genes and Genomes (KEGG), Gene Ontology (GO), non-redundancy (Nr) and Nucleotide Sequence Database (Nt), EuKaryotic Orthologous Groups (KOG), Protein family (Pfam), Clusters of Orthologous Groups (COG) and Swiss-Prot database were blasted to obtain annotation information and functional classification of unigene with *e*-value <10^−5^ (Altschul et al., 1990; Nawrocki et al., 2009; Buchfink et al., 2015). We compared clean reads to the genome sequence using Bowtie2, and then calculated the gene expression levels of each sample using RSEM (a software package for calculating gene expression levels of RNA-seq reads and transcription subtypes). The Pearson correlation coefficient between each two samples was calculated using cor function in R software. Principal component (PCA) analysis was performed using princomp function in R software. DESeq software was used to detect deg between the control group and the experimental group (Love et al., 2014).

### 2.8 GO and KEGG analysis

GO can be divided into three functional categories: molecular function, cellular component and biological process. Classify according to the difference of gene detection function, at the same time, use R-phyper function to enrich and analyze in the software, calculate *p*-value, and then take the function of FDR correction *p*-value usually ≤0.05 as significant enrichment. According to KEGG annotation results and official classification, we classified the differentially expressed genes into biological pathways. At the same time, we used the phyper function in R software for enrichment analysis and calculated the *p*-value. The *p*-value was then FDR corrected, and in general, functions with *q* value ≤0.05 were considered significantly enriched.

### 2.9 Identification of P450 and CarE genes and RT-qPCR

The P450 and CarE genes were searched according to annotation information in the transcriptome of *S. heraclei*. The repetitive sequences were removed, and the amino acid sequences of the remaining P450 and CarE genes were BLAST compared with the Nr database of NCBI (National Center for Biotechnology Information) to remove misannotated amino acid sequences.

Reverse transcription from 1 μg total RNA to cDNA was performed using the StarScript III All-in-one RT kit with gDNA Remover (Genstar, China). The RT-qPCR was detected via LineGene 9,600 Plus real-time PCR detection system (BIOER, China) with 2×RealStar Fast SYBR qPCR Mix (Low ROX) (Genstar, China). *β-Tubulin* was used as the endogenous control, and the 2^−ΔΔCT^ method was used to calculate the relative expression (Livak and Schmittgen, 2001; Ma et al., 2016). The RT-qPCR reaction consisted of three independent technical and biological replicates. Primers were listed in Supplementary Table S1.

### 2.10 RNA interference

The T7 high yield transcription kit (Invitrogen, United States) was used to synthesize the double-stranded RNA (dsRNA) of *CYP6DA2*, *CarFE4* and enhanced green fluorescent protein (*EGFP*). The primers were listed in Supplementary Table S1. The dsRNA of *CYP6DA2*, *CarFE4* and *EGFP* was added to artificial diet (sterile sucrose solution, 0.5 M) at a concentration of 150 ng/μL. The third-instar nymphs of *S. heraclei* were transferred onto the artificial diet containing dsRNA of *CYP6DA2* and *CarFE4* for rearing 2 days. The surviving insects were collected for detection of interference efficiency. The third-instar nymphs of *S. heraclei* feeding on artificial diet containing dsRNA of *EGFP* were as a control. After dsRNA feeding 1 day, the third-instar nymphs of *S. heraclei* were treated with afidopyropen at the dose of LD_50_ (Liu Y. X. et al., 2024). The mortality was checked in 48 h. Each treatment was repeated three times and contained thirty insects.

### 2.11 Statistical analysis

The statistical analysis was performed via SPSS 20.0 software (IBM Corporation). The Student’s *t*-test and Tukey’s multiple comparison test were used to compare the differences between treatments. Data were shown as mean ± standard error (SE). The *p* < 0.05 and *p* < 0.01 were considered statistically significant and very significant differences, respectively.

## 3 Results

### 3.1 Toxicity of afidopyropen to *S. heraclei*

After 48 h treatment, the LD_50_ value of afidopyropen was 0.050 ng/pest (Table 1). In further experiments, the LD_50_ dose of afidopyropen was used to evaluate its effect on *S. heraclei*.

**TABLE 1:** The toxicity of afidopyropen to *S*. *heraclei*.

Insecticide	*n*	LD_50_ (ng/pest) (95% CI)	Slope ± SE	χ2 (df)
Afidopyropen	417	0.050 (0.039–0.069)	1.261 ± 0.175	4.162 (3)

*n*, number of insects assayed. CI, confidence interval; SE, standard error. χ2, Chi-square value. df, degrees of freedom.

### 3.2 Effect of afidopyropen on the detoxification enzymes of *S. heraclei*

**FIGURE 1:**
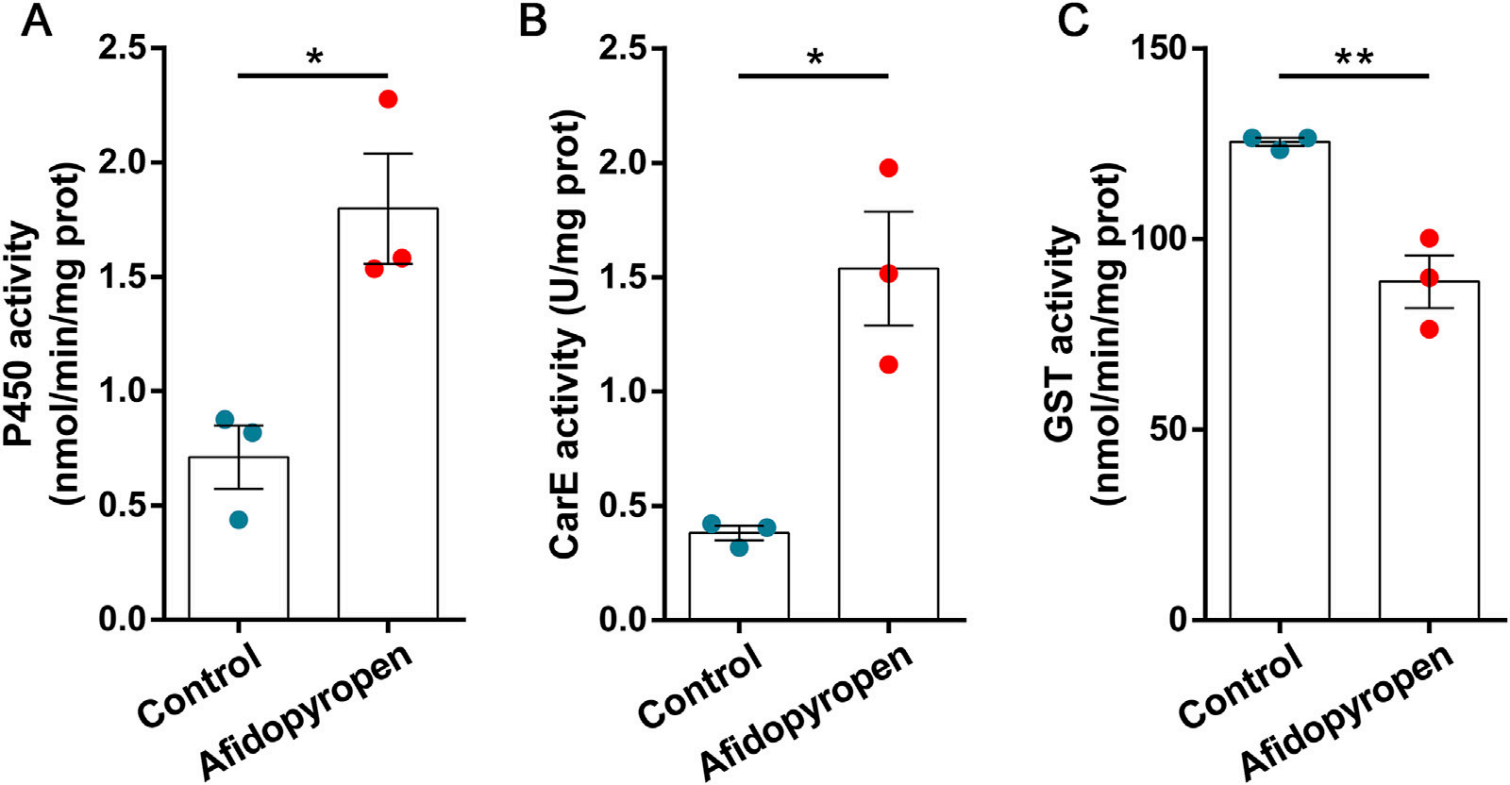
Detoxification enzyme activity in *S. heraclei* after afidopyropen treatment. **(A)** MFO activity after treatment of afidopyropen. **(B)** CarE activity after treatment of afidopyropen. **(C)** GST activity after treatment of afidopyropen. The asterisk (*) and asterisks (**) indicate significant differences with *p* < 0.05 and *p* < 0.01 compared to control group.

The relative level of P450 activity in *S. heraclei* was remarkably increased by 2.60-fold under afidopyropen treatment at 48 h compared to the control (Figure 1A). The relative level of CarE activity was remarkably increased by 4.86-fold under afidopyropen treatment at 48 h compared to the control (Figure 1B). The relative level of GST activity was significantly decreased under afidopyropen treatment at 48 h compared to the control (Figure 1C).

### 3.3 Synergistic effects of PBO, TPP and DEM on the mortality of *S. heraclei*

**FIGURE 2:**
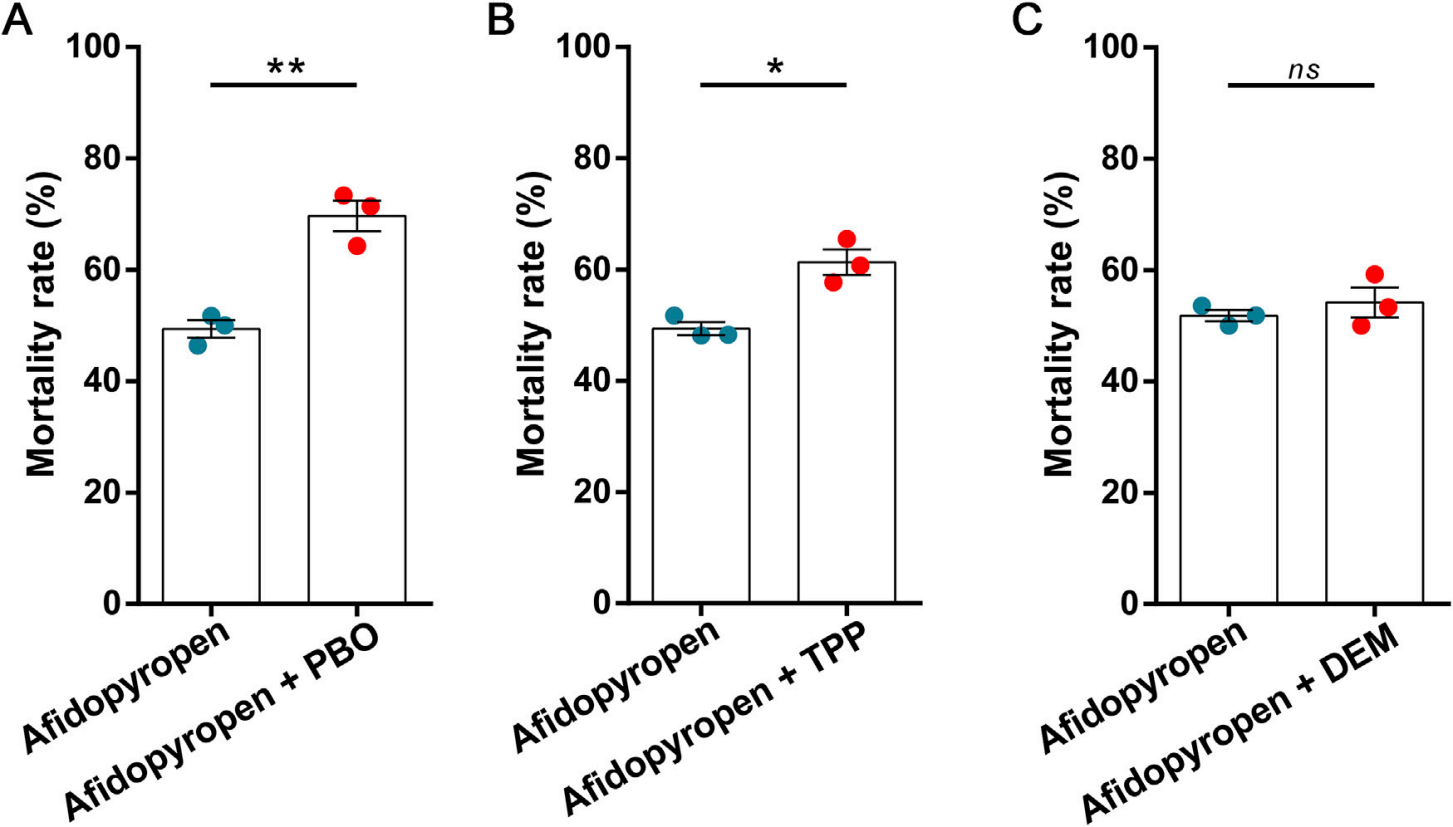
Effects of synergists on mortality of *S. heraclei* under afidopyropen treatment. **(A)** The synergism of PBO on *S. heraclei* mortality after treatment of afidopyropen. **(B)** The synergism of TPP on *S. heraclei* mortality after treatment of afidopyropen. **(C)** The synergism of DEM on *S. heraclei* mortality after treatment of afidopyropen. The asterisk (*) and asterisks (**) indicate significant differences with *p* < 0.05 and *p* < 0.01 compared to afidopyropen group. The *ns* represents no significant difference.

After PBO treatment, the mortality of *S. heraclei* was significantly increased by 1.41-fold under afidopyropen treatment compared to the control (Figure 2A). After TPP treatment, the mortality of *S. heraclei* was significantly increased by 1.24-fold under afidopyropen treatment compared to the control (Figure 2B). After DEM treatment, there was no significant synergistic effect on the mortality of *S. heraclei* treated with afidopyropen compared to the control (Figure 2C).

### 3.4 Overall analysis of transcriptome data

A total of six samples were sequenced, yielding an average of 21, 925, 000 clean reads. The clean reads ratio was greater than 95.00%. The data quality evaluation showed that the Q20 values of the six samples were greater than 96.00%, and the Q30 values were greater than 89.50% (Supplementary Table S2), indicating good data quality. The clean reads were finally assembled into 62,844 unigenes, with an average length of 1,486 bp, N50 length 2,829 bp, N70 length 1,804 bp, and GC percentage counting range from 33.13% to 33.56% (Supplementary Table S3).

The number of unigenes in the range of sequence length from 200 nt to 300 nt was the largest (11,818), and the number of unigenes with sequence length ≥3,000 nt was the second (9,618) (Supplementary Figure S1A). In order to ensure the rationality and reliability of samples and the repeatability of biological experiment, correlation analysis and principal component analysis were performed on six samples before differential gene analysis. The correlation coefficients of the six samples were all above 96.80%, indicating that the gene expression levels of the six samples were very similar (Supplementary Figure S1B). The principal component analysis demonstrated that the percentage value of PC1 was 98.71% and PC2 was 1.04%. This indicates that the repeatability of intra-group samples was good, the data similarity was high, the discrimination between groups was good, and there were no outlier samples (Supplementary Figure S1C).

### 3.5 Functional annotation and analysis of differentially expressed genes (DEGs)

**FIGURE 3:**
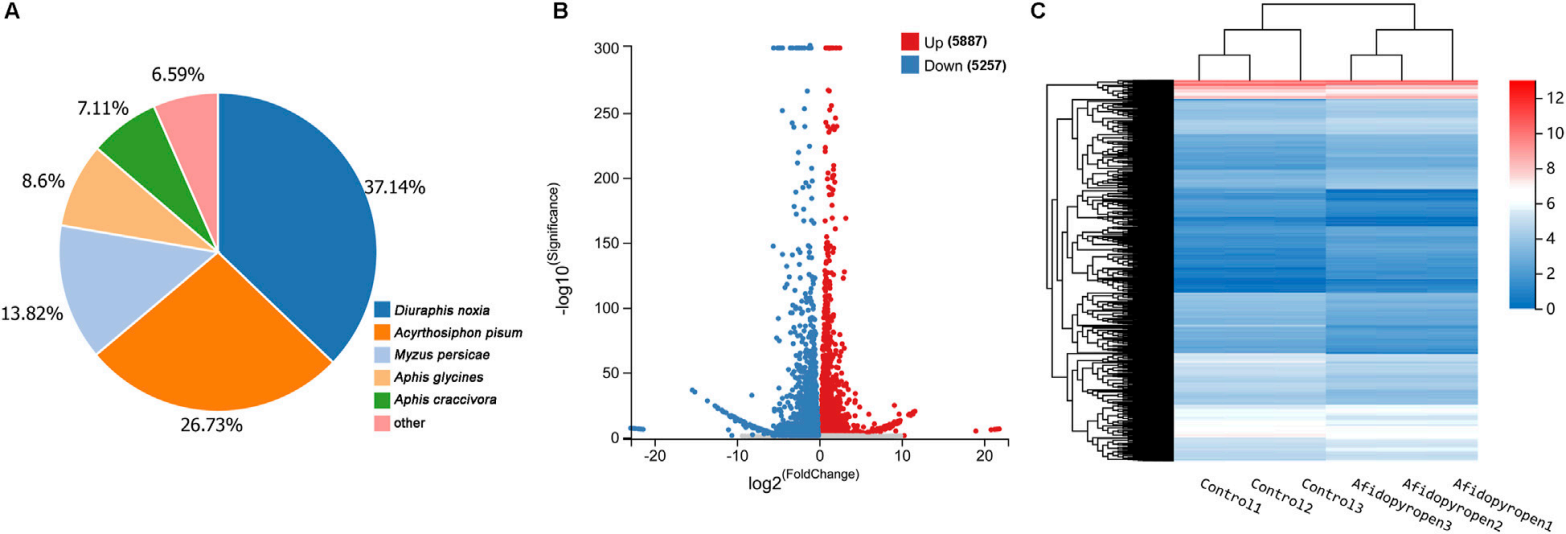
Differential gene expression analysis of *S. heraclei* in response to afidopyropen treatment. **(A)** Species distributions of top blastx matches to Nr database. **(B)** Number of up- and downregulated DEGs and volcano plot of DEGs in afidopyropen exposure. **(C)** Heatmap of DEGs between afidopyropen and control.

Through the Blastx homology search, a total of 12,600 unigenes matched entries in the NR database. Most of them shared homology with Hemiptera, such as *Diuraphis noxia* (37.14%), *Acyrthosiphon pisum* (26.73%), *M. persicae* (13.82%), *Aphis glycines* (8.60%) and *Aphis craccivora* (7.11%) (Figure 3A). To identify DEGs associated with afidopyropen treatment, DESeq method was used to analyze the DEGs between control and treatment group samples, and a total of 11,144 DEGs were detected, of which 5,887 were upregulated and 5,257 were downregulated (Figure 3B). A heat map of differential gene expression showed a clear visual comparison of DEGs under afidopyropen treatment (Figure 3C).

To further elucidate the biological functions and metabolic pathways of 11,144 DEGs, the biological processes and metabolic pathways of these unigenes were classified using the GO database and KEGG database. According to the GO analysis results, a total of 23 biological processes, 14 cell components and 9 molecular function catalogues were gathered. In biological processes, most DEGs were involved in “cellular process” (3,120), “metabolic process” (2,513), “biological regulation” (1,938) and “biological regulation” (1,785). In the cell component, the number of “cells” was 3,350, the number of “cell parts” was 3,350 and the number of “organelle” was 2,762. In terms of the molecular function catalogs, most GO terms were involved in “binding” (2,803) and “catalytic activity” (1,908) (Supplementary Figure S2A). These data indicated that many biological processes, organelles, and molecular functions were involved in the response of *S. heraclei* to afidopyropen. KEGG pathway analysis, according to the results of these unigenes involved five metabolic pathways, including Cellular the Processes, Environmental Information Processing, Genetic Information Processing, Metabolism and Organismal Systems. In the end, a total of 1,605 unigenes related to metabolism, including Amino acid metabolism, Biosynthesis of other secondary metabolites, Carbohydrate metabolism, Energy metabolism, Global and overview maps, Glycan biosynthesis and metabolism, Lipid metabolism, Metabolism of cofactors and vitamins, Metabolism of other amino acids, Metabolism of terpenoids and polyketides, Nucleotide metabolism, and Xenobiotics biodegradation and metabolism (Supplementary Figure S1B). Analysis of the top 20 KEGG pathways showed that the metabolism was significantly enriched in seven pathways including “Biosynthesis of amino acids”, “Selenocompound metabolism”, “Nitrogen metabolism”, Citrate cycle (TCA cycle)”, “Tyrosine metabolism”, “Ether lipid metabolism” and “2-Oxocarboxylic acid metabolism”. The genetic information processing was enriched in five pathways including “Ribosome”, “Nucleocytoplasmic transport”, “Spliceosome”, “Proteasome” and “Ubiquitin mediated proteolysis”. The cellular processes pathway was enriched in four pathways including “Apoptosis”, “Apoptosis–fly”, “Mitophagy–animal” and “Autophagy–animal”. The organismal systems pathway was enriched in three pathways including “Antigen processing and presentation”, “Renin secretion” and “Fat digestion and absorption”. The environmental information processing was enriched in one pathway including “Hedgehog signaling pathway–fly” (Supplementary Table S4).

### 3.6 Identification and screening of differentially expressed P450 and CarE genes

A total of sixteen P450 genes were identified in the transcriptome of *S. heraclei*, including four in Mito clade, six in CYP2 clade, five in CYP3 clade and one in CYP4 clade. In addition, three CarE genes were identified, including *CarFE4*, *CarE6* and *CarE4* (Table 2).

**TABLE 2:** Summary of the P450 and CarE genes identified from the transcriptome of *S*. *heraclei*.

Name	Gene Id	Identification	ORF (bp)	Species	Acc. Number	Score	*E*-value
P450s (Mito)	Unigene9860-S1	*CYP301A1*	1,579	*Acyrthosiphon pisum*	XP_001948959.2	1,056	0.00
	Unigene16067-S1	*CYP302A1*	1,510	*Diuraphis noxia*	XP_015365256.1	992	0.00
	Unigene29672-S5	*CYP314A1*	1,561	*Aphis craccivora*	QQL12330.1	949	0.00
	Unigene21790-S3	*CYP315A1*	1,360	*Diuraphis noxia*	XP_015380369.1	874	0.00
P450s (CYP2)	Unigene34833-S2	*CYP15A1*	1,483	*Sitobion avenae*	QRY28570.1	874	0.00
	Unigene9273-S4	*CYP18A1*	1,540	*Diuraphis noxia*	XP_015366692.1	1,053	0.00
	Unigene32818-S3	*CYP303A1*	1,501	*Diuraphis noxia*	XP_015370810.1	940	0.00
	Unigene37168-S1	*CYP305E1*	1,450	*Diuraphis noxia*	XP_015375411.1	988	0.00
	Unigene13207-S4	*CYP306A1*	1,480	*Diuraphis noxia*	XP_015366684.1	973	0.00
	Unigene11373-S6	*CYP307A2*	1,651	*Diuraphis noxia*	XP_015369329.1	1,011	0.00
P450s (CYP3)	Unigene856-S6	*CYP6DA2*	1,369	*Rhopalosiphum padi*	XP_060849315.1	853	0.00
	Unigene23366-S4	*CYP6CY77*	1,543	*Myzus persicae*	XP_022172415.1	1,007	0.00
	Unigene9368-S3	*CYP6DD1*	1,540	*Diuraphis noxia*	XP_015366629.1	978	0.00
	Unigene30067-S3	*CYP6YC1*	1,435	*Diuraphis noxia*	XP_015372631.1	829	0.00
	Unigene35198-S6	*CYP6DB1*	1,543	*Diuraphis noxia*	XP_015368356.1	1,047	0.00
P450s (CYP4)	Unigene24630-S4	*CYP4CH1*	1,579	*Diuraphis noxia*	XP_015369452.1	1,013	0.00
CarEs	Unigene16527-S4	*CarFE4*	1,699	*Myzus persicae*	XP_022165543.1	1,079	0.00
	Unigene22518-S6	*CarE6*	1,696	*Diuraphis noxia*	XP_015373999.1	1,100	0.00
	Unigene16516-S4	*CarE4*	646	*Metopolophium dirhodum*	XP_060878842.1	347	5E-115

**FIGURE 4:**
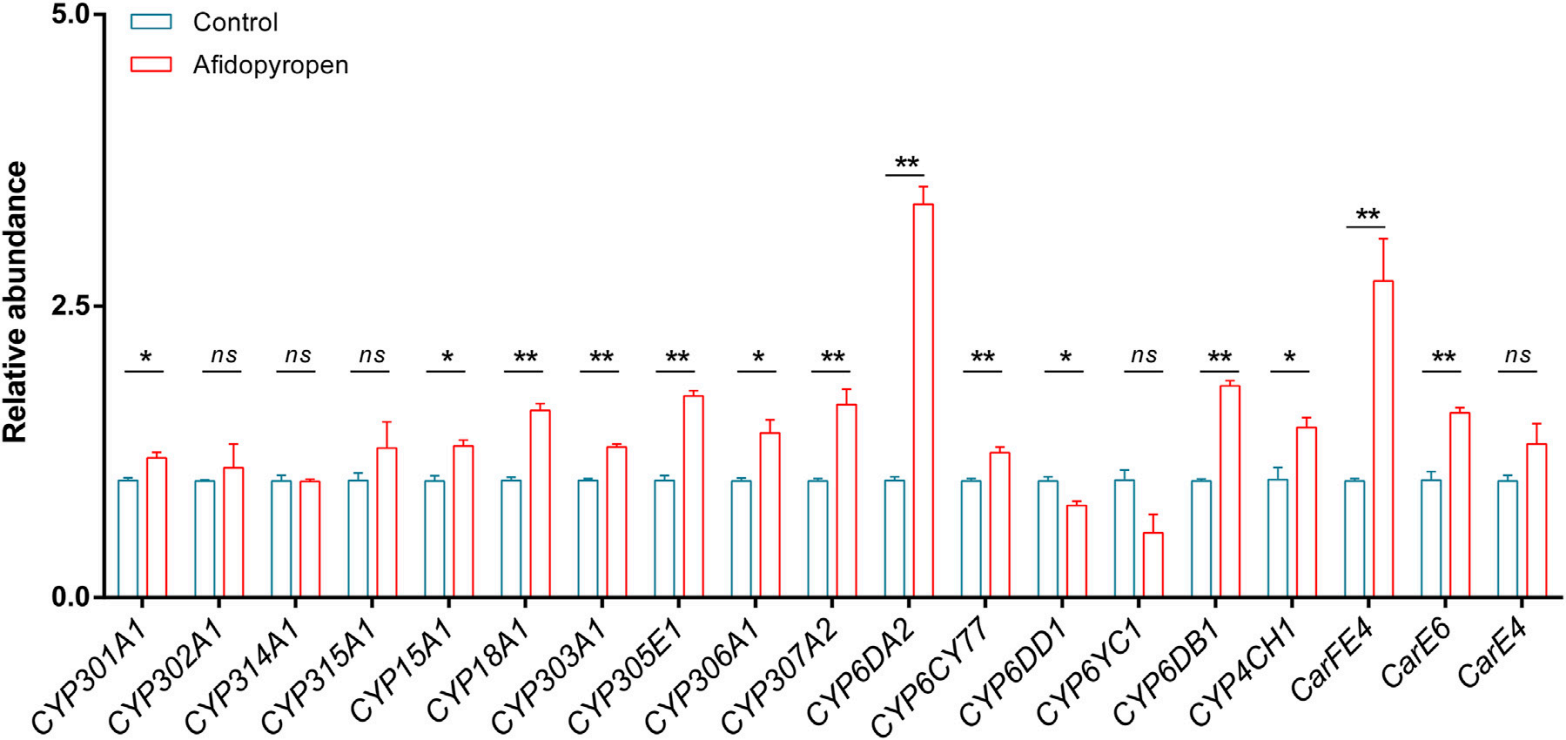
Screening of differentially expressed P450 and CarE genes by RT-qPCR analysis. The asterisk (*) and asterisks (**) indicate significant differences with *p* < 0.05 and *p* < 0.01 compared to control group. The *ns* represents no significant difference.

To identify potential P450 and CarE genes which involved in afidopyropen susceptibility, we examined the expression induction of sixteen P450 genes and three CarE genes using RT-qPCR after afidopyropen treatment. After afidopyropen treatment, eleven P450 genes were significantly upregulated, including one in Mito clade, six in CYP2 clade, three in CYP3 clade and one in CYP4 clade, among which *CYP6DA2* in CYP3 clade was the most significant (Figure 4). Two CarE genes *CarFE4* and *CarE6* were significantly upregulated, of which *CarFE4* was the most significant (Figure 4).

In the transcriptome of *S. heraclei*, the expression of up-regulated P450 genes included *CYP18A1*, *CYP306A1*, *CYP307A2*, *CYP6DA2*, and *CYP6CY77*, and the expression of downregulated P450 genes included *CYP301A1* and *CYP303A1* (Supplementary Table S5). There was no difference in the transcriptome for the CarE genes.

### 3.7 *CYP6DA2* and *CarFE4* affect the susceptibility of *S. heraclei* to afidopyropen

**FIGURE 5:**
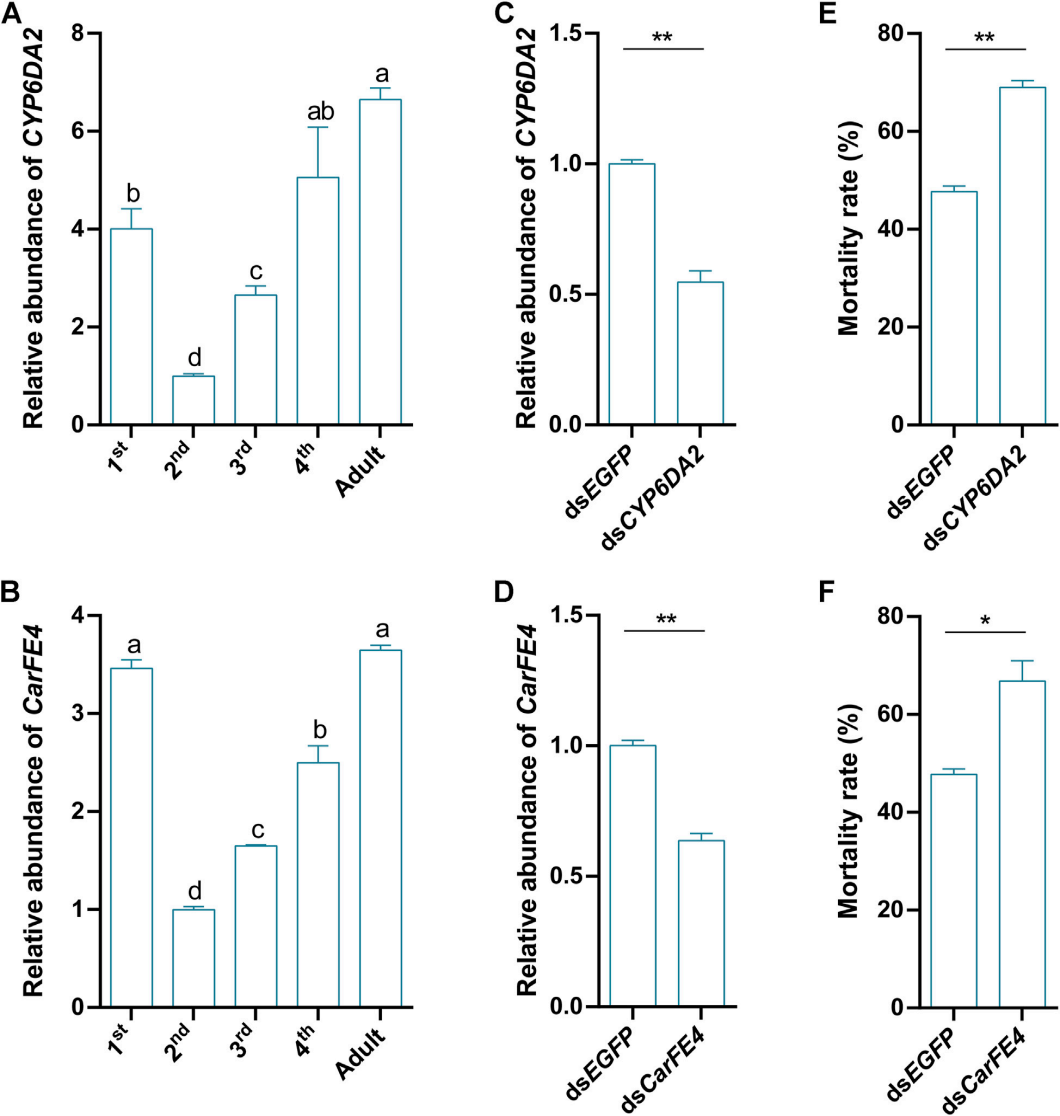
Functional analysis of *CYP6DA2* and *CarFE4* by RNAi and their expression patterns in *S. heraclei*. **(A)** The expression patterns of *CYP6DA2* at different developmental stages. **(B)** The expression patterns of *CarFE4* at different developmental stages. **(C)** Relative abundance of *CYP6DA2* in *S. heraclei* at 2 days after feeding dsRNA. **(D)** Relative abundance of *CarFE4* in *S. heraclei* at 2 days after feeding dsRNA. **(E)** Mortality rate of *S. heraclei* fed *dsCYP6DA2* treated with LD_50_ concentrations of afidopyropen for 48 h. **(F)** Mortality rate of *S. heraclei* fed *dsCarFE4* treated with LD_50_ concentrations of afidopyropen for 48 h. The asterisk (*) and asterisks (**) indicate significant differences with *p* < 0.05 and *p* < 0.01 compared to control group. The letters a, b, c, and d present significant differences (*p* < 0.05) of the expression.

To further explore the functions of *CYP6DA2* and *CarFE4*, we examined the expression of these two genes in *S. heraclei* at different developmental stages after treatment with afidopyropen. The results demonstrated that *CYP6DA2* and *CarFE4* were expressed in all five stages, from the first-instar larvae to the adult stage, following treatment with the drug. The highest expression of *CYP6DA2* was observed in the adult stage, followed by the fourth-instar larvae stage. The expression of *CYP6DA2* was observed to be relatively low in the second and third-instar larval stages, with the lowest expression observed in the second-instar stage (Figure 5A). The highest levels of *CarFE4* expression were observed in the adult stage, followed by the first and fourth-instar stages. Conversely, relatively low levels of *CarFE4* expression were noted in the second and third-instar stages, with the lowest levels observed in the second-instar stage (Figure 5B). To verify the role of *CYP6DA2* and *CarFE4* in the sensitivity of *S. heraclei* to afidopyropen, we disrupted these two genes, and the results showed that the relative abundance of *CYP6DA2* and *CarFE4* (Figures 5C, D) genes was significantly reduced in the disrupted *S. heraclei* compared with the ds*EGFP* group. Further bioassay results indicated that downregulation of *CYP6DA2* and *CarFE4* (Figures 5E, F) expression significantly increased the mortality of *S. heraclei* after afidopyropen treatment.

## 4 Discussion

At present, chemical control is an important mean to manage aphids, which is simple and fast, but with the repeated use of chemical insecticides, insecticide residues and insecticide resistance have gradually become a major challenge to be solved (Silva et al., 2012; Field et al., 2017). Afidopyropen is a highly targeted and environmentally friendly insecticide with no cross-resistance to other insecticides (Ding et al., 2024), and is an important tool for integrated field management or resistance management of piercing-sucking mouthparts pests such as aphids, whitefly and leafhoppers. Understanding the detoxification mechanism of afidopyropen by pests is very important for the scientific use of afidopyropen in the field.

Metabolic resistance is one of the main mechanisms of insecticide resistance in field pests (Hemingway, 2000). The enhanced activity of detoxifying enzymes is an important line of defense against insecticides, and the main enzymes involved are P450, CarE and GST (Paula et al., 2020). Previous studies have shown that single insecticide could not induce the activity of all detoxification enzymes, such as P450 and CarE were involved in the detoxification process of *A. gossypii* to many insecticides, while GST was selectively resistant to different insecticides (Shi et al., 2023), and the activity of detoxification enzyme of *Bemisia tabaci* was different in different host plants (Guo et al., 2014). In addition, study has shown that the enhancement of P450 and CarE activity was closely related to the multiple resistance of *Laodelphax striatellus* (Gao et al., 2008). At the same time, due to the different metabolic pathways of P450 and CarE in insects and mammals, the toxicity was different, which also reflected the selective function of insecticides (Liu X. et al., 2024). The results of this study showed that after afidopyropen treatment, the activities of P450 and CarE were significantly increased, while the activity of GST was decreased. We speculated that the P450 and CarE enzymes might be involved in the detoxification process of *S. heraclei* to afidopyropen, and the direct relationship and detoxification mechanism between them were verified by synergism bioassay, but we could not deny the function of GST, which acted as a detoxification enzyme in phase II, played a late role in the detoxification process. For example, the activity of GST in *Nilaparvata lugens* was detected only 72 h after insecticide treatment (Yang B. et al., 2020). The decrease in GST activity, may be due to the fact that GST does not possess selective resistance to afidopyropen, whereas aphids, when attacked by insecticides, adjust their resources in order to survive by prioritizing the assurance of other, more critical physiological processes, with a corresponding decrease in the investment in non-emergency defense mechanisms such as GST (King and MacRae, 2015). Furthermore, it is possible that excessive oxidative stress induced by insecticides leads to a decrease in the stability of GST proteins (Hao et al., 2021; Dong et al., 2024). Chemicals and host plants affected the activity of detoxification enzymes. Different enzymes involved in the detoxification process reflected the multiplicity of insect resistance mechanisms, and the joint action of P450 and CarE might also reflect the selective insecticidal characteristics of afidopyropen. Furthermore, the metabolism of enzymes affected the efficacy of insecticides (Khan, 2020; Shakya et al., 2022), and the expression of regulatory enzymes could restore or maintain the sensitivity of insects to insecticides to a certain extent.

Transcriptome is a technology for analyzing transcripts to reveal the regulation of gene expression and to annotate its biological function (Jones et al., 2024). In the transcriptome data of this study, we found seventeen P450 genes and three CarE genes. The number of P450 and CarE genes is relatively low. This may be related to the nature of exogenous substances (Katsavou et al., 2022), the true sociality of insect species (Zhang et al., 2023), the adaptability of host plants and survival environments (Li Y. et al., 2020; Lu et al., 2023), and even the influence of sequencing techniques on transcriptional data (Yang Y. X. et al., 2020). P450 genes are divided into several families, among which CYP3 and CYP4 families are related to the detoxification metabolism of insecticides. CYP3 family is even more important genes involved in the detoxification metabolism that are unique to insects (Feyereisen, 2012; Han et al., 2022). Quantitative detection of P450 and CarE genes showed that thirteen genes were up-regulated after afidopyropen treatment, of which one-third belonged to CYP3 and CYP4 family, and the expression of *CYP6DA2* and *CarFE4* was upregulated by more than 2.5-fold. *CYP6DA2* was significantly expressed in *A. gossypii* (Peng et al., 2016) under insecticides exposure, which was consistent with our results. *CarFE4* was involved in the resistance mechanism of insects and would respond to external adverse factors (Ma et al., 2018). Under the selection of insecticides, *M. persicae* with low *CarFE4* gene level were gradually replaced by *M. persicae* with high level of *CarE4* gene (Field and Foster, 2002). At the same time, the amplification of *CarFE4* gene made *M. persicae* resistant to a variety of insecticides in the field (Tang et al., 2017). Based on this, we have reason to speculate that *CYP6DA2* and *CarFE4* play an important role in the insecticide detoxification in insects.

At the same time, the number of genes obtained from the transcriptome is so large that it is impossible to analyze its function alone, so in the course of the experiment, genes with similar functions are grouped together to form pathways, so as to realize the correlation between function and phenotype (Li et al., 2022; Kakino et al., 2023). The GO and KEGG annotations in this study showed some of the most representative biological functions and information processing pathways. The analysis of these results revealed that the pathways related to higher concentration of differentially expressed genes, mainly included carbohydrate metabolism, energy metabolism, lipid metabolism, amino acid metabolism and so on. These metabolic pathways played an important role in the daily actions, growth and development of insects (Jiang et al., 2020; Xu et al., 2022). After afidopyropen treatment, the genes in these metabolic pathways were enriched, and it was speculated that *S. heraclei* achieved metabolic detoxification of insecticides through these pathways. A large amount of energy will be generated in the process of Metabolism to provide enough power for life activities, and a large amount of reactive oxygen species (ROS) will be generated at the same time. Previous studies have reported that ROS can regulate detoxification metabolism related genes through CncC pathway to enhance the resistance of *Bactrocera dorsalis* to *β*-cypermethrin (Zeng et al., 2024). In addition, signal transduction was also a relatively enriched pathway of differentially expressed genes, which regulated various physiological activities throughout the insect’s life and was an extremely important mechanism in the organism (Meng et al., 2019), and we speculated that it also regulated the detoxification process of *S. heraclei* to afidopyropen.

In order to further verify the functions of *CYP6DA2* and *CarFE4*, we need to silence these two genes by RNAi technology (Zhou et al., 2021), and then observe the effect of afidopyropen on *S. heraclei*. Because of its simplicity and good control effect, RNAi has been widely used in gene function identification. For example, in the experiment of resistance of *A. gossypii* to plant defense substances, the mortality of *A. gossypii* was significantly increased by silencing related genes by RNAi (Ma et al., 2017). The main methods of RNAi administration are microinjection and artificial feeding. Although the artificial feeding method has its own determinations, such as the inability to accurately determine the feeding habits of insects (Jain et al., 2020), many Hemiptera insects have successfully studied the function of key genes, such as *Sitobion avenae* (Fan et al., 2015), *M. persicae* (Tariq et al., 2019), *B. tabaci* (Vyas et al., 2017), *N. lugens* (Chen et al., 2010) and so on, so the feasibility of feeding is also very high. The interference results of this study showed that the silencing of *CYP6DA2* and *CarFE4* could significantly increase the susceptibility of *S. heraclei* to afidopyropen. The results suggested that *CYP6DA2* and *CarFE4* were indeed involved in the regulation of the detoxification process of *S. heraclei* and played a key role in reducing the susceptibility of insecticides.

In order to determine the critical period of the control effect of afidopyropen on *S. heraclei*, the expression of *CYP6DA2* and *CarFE4* in *S. heraclei* at different developmental stages was detected. It has been proved that the cuticle of insects played an important role in resisting insecticides (Yan et al., 2022), and the microbiota of insects also protected insects at different developmental stages in the face of external stimuli (Huang et al., 2019). The activity of insect detoxification enzymes increased with the increase of insect development (Durak et al., 2021), which meant that with the gradual development of insects, their susceptibility to insecticides would gradually decrease. The reason for choosing the third instar nymph for most of the insect experiments is that at this time, the insects are physiologically more mature and more sensitive to environmental changes, making it easier to observe the effects of specific treatments on the insect (Kim and Roberts, 2012; Durak et al., 2021; Pers and Hansen, 2021). The body size is moderate, which is convenient for experimental operation, and it begins to show more obvious activity behavior, which is convenient for observing its behavioral pattern. Meanwhile, in the entomological study of Hemiptera, the third-instar nymph stage has been widely studied and experienced (Fan et al., 2020). The results showed that the expression of *CYP6DA2* and *CarFE4* related to detoxification was the lowest in the second- and third-instar nymphs and the highest in adults, indicating that the activity of detoxification enzyme in the second- and third-instar nymphs was low, at this time, *S. heraclei* was weak in detoxification ability to insecticides, and the control effects in these two periods might be better than those in other periods.

## 5 Conclusion

In conclusion, the results of this study can lay a foundation for further exploring the detoxification mechanism of *S. heraclei* on afidopyropen. It will also provide a theoretical basis for the rational use of afidopyropen to control aphids in the field.

## Data Availability

The datasets presented in this study can be found in online repositories. The names of the repository/repositories and accession number(s) can be found below: https://www.ncbi.nlm.nih.gov/, GSE272830: https://www.ncbi.nlm.nih.gov/geo/query/acc.cgi?acc=GSE272830.
