# Supplementary material for: Two detoxification enzyme genes, CYP6DA2 and CarFE4, mediate the susceptibility to afidopyropen in Semiaphis heraclei
Source: Front Physiol. 2024 Dec 6;15:1478869. doi: 10.3389/fphys.2024.1478869 (PMC11659293; doi:10.3389/fphys.2024.1478869)
Supplement: Supplementary file 1 [file Table1.docx]

**Table S1.** Primers used in this study.

| Gene name | Primers | Application |
| --- | --- | --- |
| *CYP301A1-F* | CAGACCTAGAAGCGACTAA | RT-qPCR |
| *CYP301A1-R* | GCCTATGACTGGTAACATTC |  |
| *CYP302A1-F* | GACCGATTACATCACAATGG |  |
| *CYP302A1-R* | AGCGTCTCTATGTCTTCTG |  |
| *CYP314A1-F* | CGTTCACTTGGCATACAA |  |
| *CYP314A1-R* | GTAGTTCGCTGTTACTTCAT |  |
| *CYP315A1-F* | AAGAAATACCAGTTGTTAAAGG |  |
| *CYP315A1-R* | GCACCATATTGTTGATGAC |  |
| *CYP15A1-F* | TCCGCATAAATATCCAATAGG |  |
| *CYP15A1-R* | AACACTTGACCGTAACATT |  |
| *CYP18A1-F* | GACGAACGGAACATACAG |  |
| *CYP18A1-R* | TATCTCCAACAGGTAGGC |  |
| *CYP303A1-F* | AGAAGCCGTTCAGTTAGT |  |
| *CYP303A1-R* | GACCATAATGTGTTCAAGACT |  |
| *CYP305E1-F* | CCTAAGTTGTTAGCATTGATGA |  |
| *CYP305E1-R* | TGTGTTCAGCGATATTATTGG |  |
| *CYP306A1-F* | ATCCTACCGAACCTTACAA |  |
| *CYP306A1-R* | AATGCCATTCTAACCTGTG |  |
| *CYP307A2-F* | GAAGTCCTCATCACCAAC |  |
| *CYP307A2-R* | CCACGACCAATCACATAG |  |
| *CYP6DA2-F* | TTACACCCACACTGAAATC |  |
| *CYP6DA2-R* | ACGATCATCCTTCTCACTT |  |
| *CYP6CY77-F* | CTATTACGAGTTGGCTGGT |  |
| *CYP6CY77-R* | TAACGGATTGGCTGTGAA |  |
| *CYP6DD1-F* | ATGTGATGCTTAGGATGAC |  |
| *CYP6DD1-R* | CAACCGAATAACTTCTGGAT |  |
| *CYP6YC1-F* | CCTTAGGTCTCCACGATG |  |
| *CYP6YC1-R* | GACGGTAAGAGGCTCATT |  |
| *CYP6DB1-F* | CAGAGCATCATTGGTTATTCT |  |
| *CYP6DB1-R* | TGTTAATTGACCTCTCATTCG |  |
| *CYP4CH1-F* | AGCAACAGGAGAATGGTAT |  |
| *CYP4CH1-R* | GGAACACTTATATCGCACTC |  |
| *CarFE4-F* | GTCCAACACTGTCGTAAGA |  |
| *CarFE4-R* | GGTGGTTTAGCATAGGGTAT |  |
| *CarE6-F* | GTCATCAGTATCCAGTAGTAGA |  |
| *CarE6-R* | TTATTGGCTGTGCTTCCT |  |
| *CarE4-F* | AATGAATGGAACACAGAAAGT |  |
| *CarE4-R* | TAACAGTAAATTGGCGTAACAT |  |
| *β-tubulin-F* | GATGAACACGGCATTGAC |  |
| *β-tubulin-R* | GGCACGAGGTACATACTT |  |
| *ds-CYP6DA2-F* | TAATACGACTCACTATAGGGGATTACGTAGTATGCCCGAC | dsRNA synthesis |
| *ds-CYP6DA2-R* | TAATACGACTCACTATAGGGTGGTATTTTAAACTTTTTGG |  |
| *ds-CarFE4-F* | TAATACGACTCACTATAGGGTGAATGTAACCTGGAATGCG |  |
| *ds-CarFE4-R* | TAATACGACTCACTATAGGGCGAAACATAAATCTTGCGAA |  |
| ds-*EGFP-F* | TAATACGACTCACTATAGGGAAGTTCAGCGTGTCCG |  |
| ds-*EGFP-R* | TAATACGACTCACTATAGGGCACCTTGATGCCGTTC |  |

**Table S2.** Summary of the transcriptome sequencing data from the control and afidopyropen treated samples.

M, million.

| Sample | Total Raw Reads (M) | Total Clean Reads (M) | Total Clean Bases (Gb) | Q20 (%) | Q30 (%) | Clean Reads Ratio (%) |
| --- | --- | --- | --- | --- | --- | --- |
| Afidopyropen1 | 22.88 | 22.13 | 6.64 | 98.78 | 94.18 | 96.72 |
| Afidopyropen2 | 21.71 | 21.21 | 6.36 | 98.66 | 93.61 | 97.70 |
| Afidopyropen3 | 22.88 | 22.05 | 6.62 | 98.75 | 94.01 | 96.37 |
| Control1 | 22.88 | 22.04 | 6.61 | 98.72 | 93.87 | 96.33 |
| Control2 | 22.88 | 22.05 | 6.62 | 98.74 | 93.99 | 96.37 |
| Control3 | 23.04 | 22.07 | 6.62 | 98.85 | 94.50 | 95.79 |

**Table S3.** Evaluation of assembly sequences.

| Sample | Total Number | Mean Length | N50 | N70 | GC (%) |
| --- | --- | --- | --- | --- | --- |
| Afidopyropen1 | 32,542 | 1,541 | 2,658 | 1,779 | 33.38 |
| Afidopyropen2 | 31,479 | 1,565 | 2,671 | 1,800 | 33.33 |
| Afidopyropen3 | 32,285 | 1,556 | 2,660 | 1,789 | 33.34 |
| Control1 | 32,857 | 1,523 | 2,619 | 1,767 | 33.13 |
| Control2 | 32,564 | 1,508 | 2,588 | 1,728 | 33.30 |
| Control3 | 32,685 | 1,479 | 2,531 | 1,695 | 33.56 |
| All-Unigene | 62,844 | 1,486 | 2,829 | 1,804 | 32.92 |

**Table S4.** The data of top 20 KEGG pathways.

| Pathway ID | Pathway name | Term gene number | Rich ratio | Q value |
| --- | --- | --- | --- | --- |
| ko03010 | Ribosome | 90 | 0.67 | 1.38E-07 |
| ko04612 | Antigen processing and presentation | 51 | 0.70 | 6.28E-05 |
| ko01230 | Biosynthesis of amino acids | 56 | 0.60 | 0.02 |
| ko03013 | Nucleocytoplasmic transport | 61 | 0.54 | 0.13 |
| ko03040 | Spliceosome | 103 | 0.51 | 0.13 |
| ko00450 | Selenocompound metabolism | 12 | 0.75 | 0.23 |
| ko04210 | Apoptosis | 54 | 0.53 | 0.23 |
| ko04214 | Apoptosis - fly | 49 | 0.54 | 0.23 |
| ko00910 | Nitrogen metabolism | 13 | 0.68 | 0.41 |
| ko00020 | Citrate cycle (TCA cycle) | 18 | 0.58 | 0.55 |
| ko00350 | Tyrosine metabolism | 11 | 0.69 | 0.55 |
| ko00565 | Ether lipid metabolism | 12 | 0.63 | 0.55 |
| ko01210 | 2-Oxocarboxylic acid metabolism | 13 | 0.62 | 0.55 |
| ko03050 | Proteasome | 18 | 0.58 | 0.55 |
| ko04120 | Ubiquitin mediated proteolysis | 74 | 0.49 | 0.55 |
| ko04137 | Mitophagy - animal | 32 | 0.54 | 0.55 |
| ko04140 | Autophagy - animal | 84 | 0.48 | 0.55 |
| ko04341 | Hedgehog signaling pathway - fly | 22 | 0.56 | 0.55 |
| ko04924 | Renin secretion | 49 | 0.51 | 0.55 |
| ko04975 | Fat digestion and absorption | 30 | 0.54 | 0.55 |

**Table S5.** The differentially expressed P450 genes in the transcriptome of *S. heraclei*.

| Name | Gene ID | Up/Down | log2^fcControl-vs-Afidopyropen^ | Q value | P value |
| --- | --- | --- | --- | --- | --- |
| *CYP301A1* | Unigene9860-S1 | Down | -1.14 | 2.34E-23 | 5.02E-25 |
| *CYP18A1* | Unigene9273-S4 | Up | 0.83 | 1.28E-21 | 2.97E-23 |
| *CYP303A1* | Unigene32818-S3 | Down | -0.80 | 9.48E-30 | 1.56E-31 |
| *CYP306A1* | Unigene13207-S4 | Up | 0.56 | 0.007937 | 0.0011897 |
| *CYP307A2* | Unigene11373-S6 | Up | 1.22 | 6.92E-17 | 2.13E-18 |
| *CYP6DA2* | Unigene856-S6 | Up | 1.22 | 0.001672 | 0.0002118 |
| *CYP6CY77* | Unigene23366-S4 | Up | 0.46 | 0.006575 | 0.0009615 |
