# Supplementary figures and images for: Two detoxification enzyme genes, CYP6DA2 and CarFE4, mediate the susceptibility to afidopyropen in Semiaphis heraclei
Source: Front Physiol. 2024 Dec 6;15:1478869. doi: 10.3389/fphys.2024.1478869 (PMC11659293; doi:10.3389/fphys.2024.1478869)

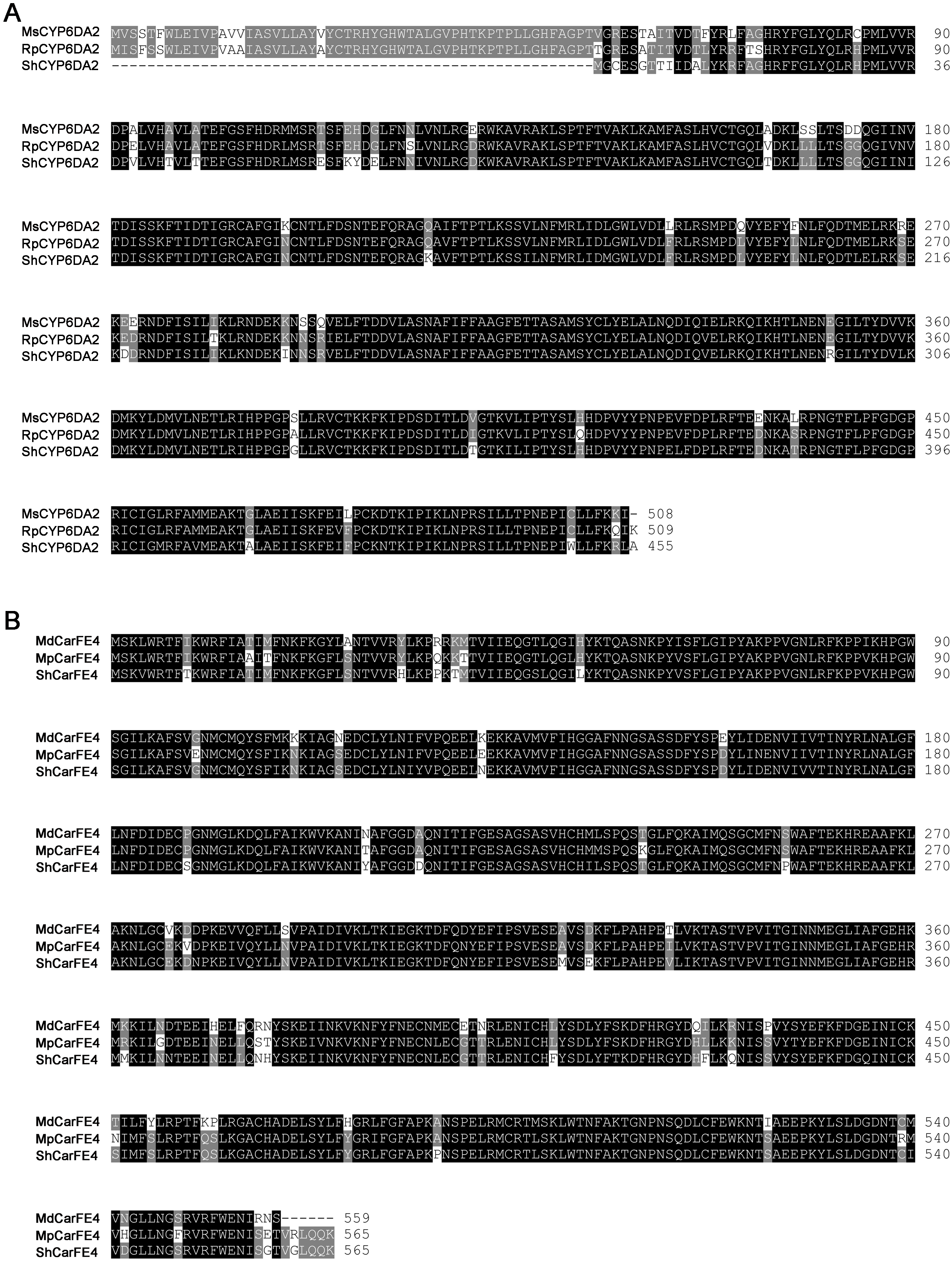

Supplement: Supplementary file 2 [file Image3.tif]

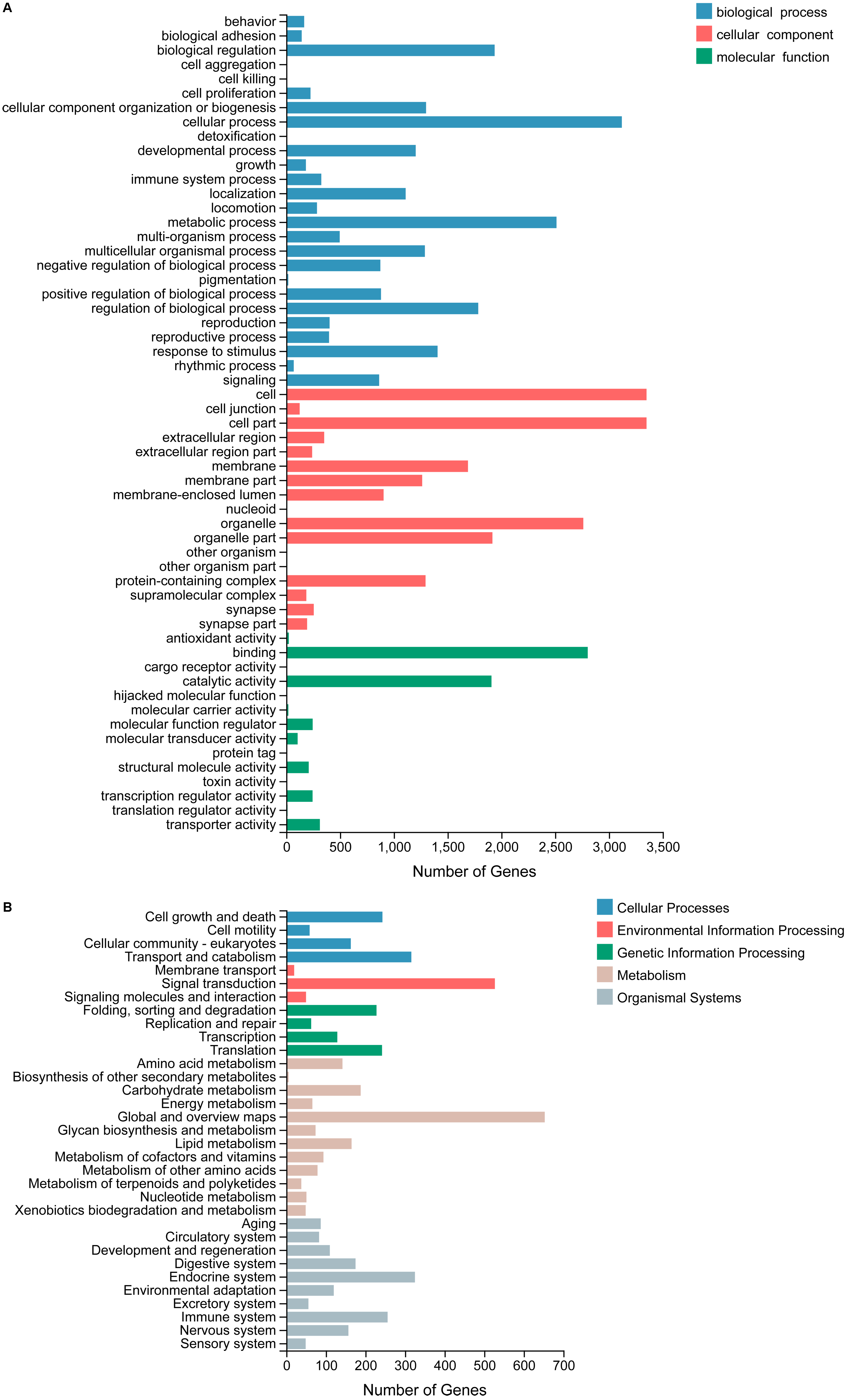

Supplement: Supplementary file 3 [file Image2.tif]

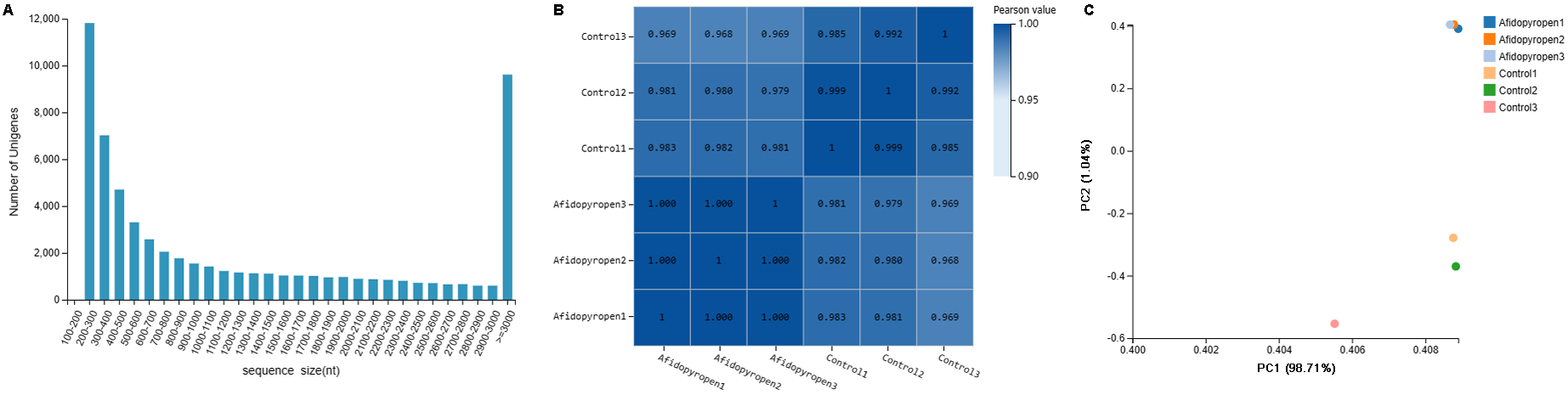

Supplement: Supplementary file 4 [file Image1.tif]
